# Clinical features and management of LICATS involving the heart in a patient with diffuse and rapidly progressive systemic sclerosis

**DOI:** 10.1016/j.ero.2026.02.009

**Published:** 2026-03-09

**Authors:** Gerlando Natalello, Silvia Laura Bosello, Riccardo Marano, Enrico De Lorenzis, Eleonora Moliterno, Eugenio Galli, Sabrina Giammarco, Melanie Hagen, Daria Pagliara, Georg Schett, Franco Locatelli, Simona Sica, Maria Antonietta D’Agostino

**Affiliations:** 1 Department of Geriatrics, Orthopedics and Rheumatology, Rheumatology and Clinical Immunology Division, Fondazione Policlinico Universitario “Agostino Gemelli” IRCCS, Rome, Italy; 2 Catholic University of the Sacred Heart, Rome, Italy; 3 Department of Radiological and Haematological Sciences, Section of Radiology, Fondazione Policlinico Universitario “Agostino Gemelli” IRCCS, Rome, Italy; 4 Department of Laboratory and Hematological Sciences, Hematology and Hematopoietic Stem Cell Transplantation Unit, Fondazione Policlinico Universitario ‘Agostino Gemelli’ IRCCS, Rome, Italy; 5 Department of Internal Medicine 3–Rheumatology and Immunology, Friedrich-Alexander-Universität (FAU) Erlangen-Nürnberg and Universitätsklinikum Erlangen, Erlangen, Germany; 6 Department of Hematology/Oncology, Cell and Gene Therapy, IRCCS Ospedale Pediatrico Bambino Gesù, Rome, Italy; 7 Deutsches Zentrum Immuntherapie, Friedrich-Alexander-Universität (FAU) Erlangen-Nürnberg and Universitätsklinikum Erlangen, Erlangen, Germany

Cell therapy with CD19-targeting chimeric antigen receptor (CAR) T-cells is a promising approach for the treatment of B-cell-driven autoimmune disorders, including progressive and treatment-refractory systemic sclerosis (SSc) [[1], [2], [3]]. CD19-CAR T-cells have a remarkable capacity to kill B-cells in tissues [4], which may lead to transient inflammatory reactions in the affected tissues. Recently, Hagen et al [5] described local immune effector cell-associated toxicity syndrome (LICATS), which constitutes a time-limited worsening of the function of an organ previously affected by autoimmune disease. It has been hypothesised that LICATS results from the death and inflammatory clearance of B-cells following CAR T-cell infiltration of the affected organ, which may contribute to the efficacy of the treatment observed so far.

Here we reported a challenging case of LICATS manifesting as transient myocarditis in a 55-year-old woman with progressive diffuse cutaneous SSc (positive antinuclear antibodies and antitopoisomerase I antibodies) and pre-existing cardiac involvement in the ongoing phase 1/2 CATARSIS trial (European Union Clinical Trial number 2024-511293-74-01). The patient, with early diffuse SSc, was enrolled in the CAR T-cell trial due to progressive skin, lung, and heart involvement, despite sequential therapy with methotrexate and mycophenolate mofetil plus low-to-medium-dose glucocorticoids. At baseline (BL), the patient had a modified Rodnan skin score (mRSS) of 33 points, with diffuse melanoderma and ‘salt-and-pepper’ depigmentation. Regarding the lungs, a forced vital capacity of 77% and diffusing capacity for carbon monoxide of 76% of the predicted value were found, and the chest computed tomography scan showed evidence of nonspecific interstitial pneumonia involving <20% of the lung parenchyma.

From the cardiac point of view, the electrocardiogram (ECG) showed nonspecific T-wave abnormalities, while 24-hour monitoring documented a nonsignificant number of supraventricular (n=10) and ventricular (n=6) ectopic beats. Cardiac magnetic resonance imaging (MRI) scan showed signs of mild fibrosis and fibro-adipose metaplasia of the left ventricular myocardium, in the absence of obvious signs of active myocarditis (mean native T1 1036 ms, mean extracellular volume [ECV] 33%, T2 mapping maximum <55 ms). However, the patient presented with elevated levels of high-sensitivity troponin I (TnI) level (226 ng/L; upper limit of normality [ULN] = 37 ng/L), creatine phosphokinase level (324 U/L; ULN = 145 U/L), and N-terminal pro-B-type natriuretic peptide (NT-proBNP) (165 pg/mL; ULN = 150 pg/mL).

In addition, clinical and radiological signs of mild oesophagus involvement were found, marked tendon friction rubs and arthralgias were reported, and malaise and low-grade fever were present. No history of digital ulcers was reported. Laboratory assessment highlighted the high activity of SSc, characterised by elevated C-reactive protein (CRP) levels (47.6 mg/L; ULN = 3.5 mg/L) and an increased erythrocyte sedimentation rate (68 mm/h; ULN = 30 mm/h).

The patient was treated with a single infusion of 1 × 10^6^/kg body weight autologous, second-generation, lentivirus-transduced CAR T-cells manufactured by the good manufacturing practice facility of IRCCS Ospedale Pediatrico Bambino Gesù, Rome, Italy (construct from Miltenyi Biomedicine). Before infusion, lymphodepletion (LD) was conducted with 1 g/m² cyclophosphamide and 3 × 25 mg/m² fludarabine. Immunosuppressants were stopped 4 weeks before apheresis, and residual glucocorticoids were stopped the day before starting LD. Within 24 hours after CAR T infusion, the patient developed a fever (38.8°C), indicating grade 1 cytokine release syndrome (CRS). Due to the persistence of CRS for more than 72 hours despite supportive therapy, the patient received a single infusion of tocilizumab (TCZ) at a dose of 8 mg/kg on day 3, with prompt resolution of fever. No signs of immune effector cell-associated neurotoxicity syndrome were observed.

Up to day 7, the patient showed no significant changes in TnI levels compared with the BL levels (values between 217 and 291 ng/L). Starting on day 10, the TnI level increased (559 ng/L), peaking at week 8 (1969 ng/L). A cardiac MRI scan close to the TnI peak level revealed signs of myocarditis (mean native T1 1139 ms, mean ECV 43%, mean T2 60 ms). Infectious aetiology was ruled out by extensive serological testing. At the same time, worsening of skin disease (increase of mRSS scores from 33 to 42 units) and increased musculoskeletal pain occurred. Specific cardiac symptoms such as dyspnoea, palpitations, or angina were not reported. Additionally, no clinical signs of heart failure were observed, except for mild peripheral oedema, which could have been associated with the worsening of skin thickening. Low-dose diuretics and beta-blockers were initiated in consideration of the asymptomatic cardiac involvement. In addition, methylprednisolone treatment at a dose of 20 mg/d with subsequent rapid dose tapering was initiated, and a second infusion of 8 mg/kg of TCZ was administered on day 23. As the TnI levels did not normalise, 2 g/kg of intravenous immunoglobulin (IVIG)—divided into 5 consecutive daily infusions—was administered starting on day 57. Due to a stable clinical course with no signs of haemodynamic deterioration, no further immunomodulatory therapy was administered afterwards. Tight monitoring was pursued. Starting from around week 12, TnI levels decreased and normalised by week 16. Additionally, a cardiac MRI scan showed no further acute inflammatory abnormalities by week 24. Close echocardiographic and Holter ECG monitoring throughout the follow-up did not reveal significant alterations. NT-proBNP levels returned to normal levels at week 24.

Consistent with previously reported kinetics for a similar CAR T product for patients with autoimmune disorders [2], the peak number of CAR^+^ cells detected in peripheral blood was 71 cells/µL on day 10, the first time point of disappearance of CAR^+^ cells in peripheral blood was day 28, and the first time point of CD19^+^ cells reappearance in peripheral blood was week 24.

Of note, general outcomes at 24 weeks were positive, meeting the revised Composite Response Index in Systemic Sclerosis 50 [6]. In particular, improvement of skin involvement in terms of extension, thickness, and depigmentation was noted (key clinical data are reported in the accompanying Fig). Follow-up at 9 months showed normal TnI levels and further improvement in mRSS score (19 units).

**Figure:**
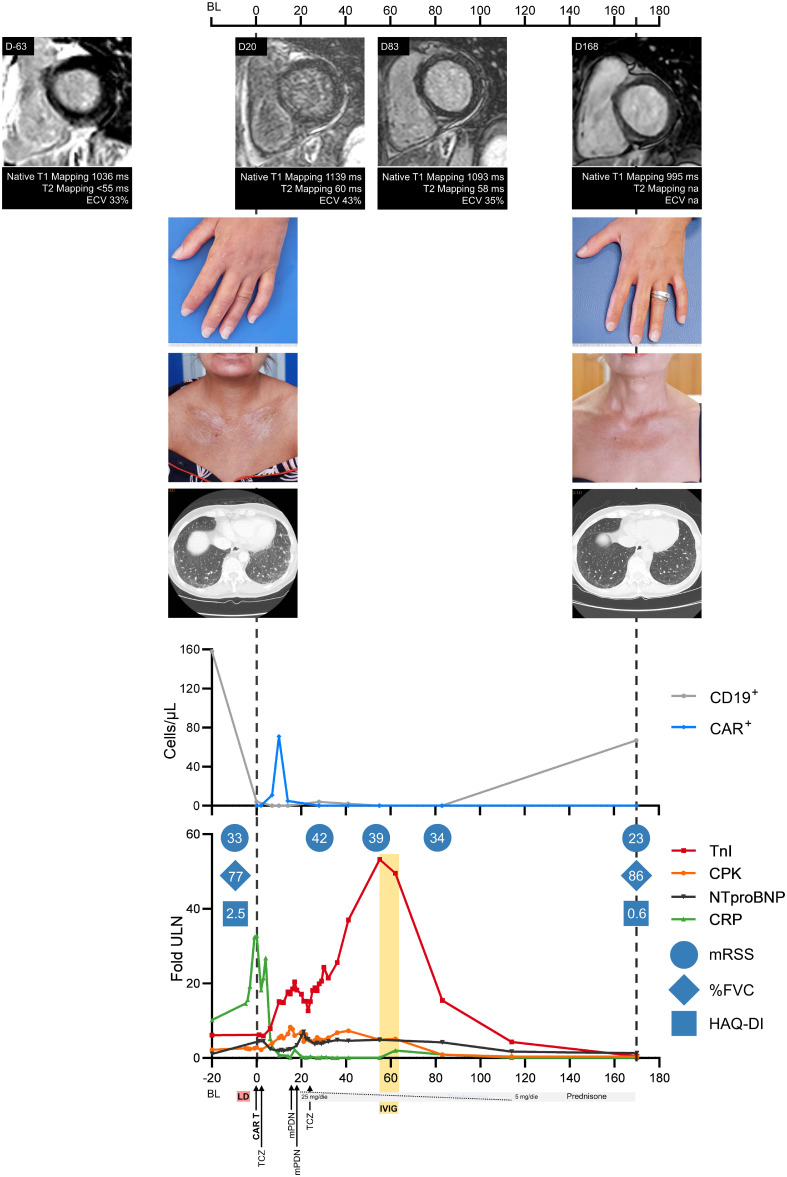
Time course in cardiac and inflammatory marker levels (fold-time increase compared with the upper limit of normal), cardiac magnetic resonance imaging data, and clinical parameters during follow-up and in relation to therapies. The x-axes show the number of days relative to CAR T infusion. BL data were collected between 2 and 4 weeks before CAR T infusion. Dose of mPDN = 20 mg. Dose of TCZ = 480 mg. Cardiac magnetic resonance imaging (MRI) scan series showing images available for evaluation of late gadolinium enhancement. Cell numbers are relative to peripheral blood assessment. BL, baseline; CAR, chimeric antigen receptor; CPK, creatine phosphokinase; CRP, C-reactive protein; ECV, extracellular volume; FVC, forced vital capacity; HAQ-DI, health assessment questionnaire-disability index; IVIG, intravenous immunoglobulin; LD, lymphodepletion; mPDN, methylprednisolone; mRSS, modified Rodnan skin score; na, not assessable; NT-proBNP, N-terminal pro-B-type natriuretic peptide; PI, postinfusion; TCZ, tocilizumab; TnI, troponin I; ULN, upper limit of normality.

This case showed LICATS grade 3 resembling myocarditis in a patient with SSc with early and progressive skin, lung, and heart involvement, elevated CRP levels, and persistent low-grade fever. She was treated at an exceptionally early stage of the disease, 9 months after disease onset, due to the severity of the disease. Notably, after exposure to CAR T-cells, the patient developed a TnI level elevation over weeks and showed changes on cardiac MRI scan. In this context, the concomitant increase in native T1 and ECV, paralleling the rise in T2 values, likely reflects the expansion of the extracellular space due to acute myocardial oedema and inflammatory infiltration, rather than the progression of fibrosis alone. However, neither heart failure nor ECG and echocardiographic abnormalities were recorded. We have chosen treatment with IVIG, as it (i) has anti-inflammatory but no immunosuppressive properties, (ii) has been used to treat myocarditis [7], and (iii) additionally compensates for the decreased immunoglobulin G levels after CD19-CAR T-cell therapy.

Overall, this case showed that LICATS can have a prolonged course in patients with severe forms of B-cell-mediated autoimmune disease treated with CAR T-cells. IVIG administration may be considered in cases of a LICATS that does not respond to glucocorticoids. In this regard, although LICATS is defined as a time-limited phenomenon that may resolve spontaneously upon the exhaustion of the immune effector cells or clearance of the target, the therapeutic contribution of IVIG was likely relevant in dampening the acute inflammatory burden. In a patient with pre-existing cardiac vulnerability, active management was prioritised to accelerate resolution and minimise the risk of adverse remodelling. Careful assessment and monitoring of the event are crucial in determining the appropriate management of patients with SSc undergoing CAR T-cell therapy. In particular, multimodal monitoring through serial assessment of cardiac biomarker levels combined with cardiovascular MRI with parametric mapping [8] appears essential for the early detection and precise characterisation of myocardial inflammation.

## CRediT authorship contribution statement

**Gerlando Natalello:** Writing – review & editing, Writing – original draft, Visualization, Methodology, Investigation, Formal analysis, Data curation, Conceptualization. **Silvia Laura Bosello:** Writing – review & editing, Writing – original draft, Methodology, Investigation, Formal analysis, Data curation, Conceptualization. **Riccardo Marano:** Writing – review & editing, Visualization, Investigation, Data curation. **Enrico De Lorenzis:** Writing – review & editing, Investigation. **Eleonora Moliterno:** Writing – review & editing, Data curation. **Eugenio Galli:** Writing – review & editing, Investigation. **Sabrina Giammarco:** Writing – review & editing, Investigation. **Melanie Hagen:** Writing – review & editing, Supervision, Methodology. **Daria Pagliara:** Writing – review & editing, Investigation. **Georg Schett:** Writing – review & editing, Writing – original draft, Supervision, Methodology, Conceptualization. **Franco Locatelli:** Writing – review & editing, Supervision, Investigation. **Simona Sica:** Writing – review & editing, Supervision, Investigation. **Maria Antonietta D’Agostino:** Writing – review & editing, Writing – original draft, Supervision, Project administration, Methodology, Investigation, Formal analysis, Data curation, Conceptualization.

## Competing interests

MAD reports receiving non-financial/in-kind support (reagents) from Miltenyi Biomedicine for this study. All other authors declare no competing interests.
